# Microfluidic channel sensory system for electro-addressing cell location, determining confluency, and quantifying a general number of cells

**DOI:** 10.1038/s41598-022-07194-4

**Published:** 2022-02-28

**Authors:** Crystal E. Rapier, Srikanth Jagadeesan, Gad Vatine, Hadar Ben-Yoav

**Affiliations:** 1 Nanobioelectronics Laboratory (NBEL), Department of Biomedical Engineering, Ben-Gurion University of the Negev, Beer Sheva, Israel; 2 The Department of Physiology and Cell Biology, Faculty of Health Sciences, The Regenerative Medicine and Stem Cell (RMSC) Research Center, The Zlotowski Center for Neuroscience, Ben-Gurion University of the Negev, Beer Sheva, Israel

**Keywords:** Electrochemistry, Lab-on-a-chip, Sensors and probes, Biomedical engineering

## Abstract

Microfluidics is a highly useful platform for culturing, monitoring, and testing biological cells. The integration of electrodes into microfluidic channels extends the functionality, sensing, and testing capabilities of microfluidic systems. By employing an electrochemical impedance spectroscopy (EIS) technique, the non-invasive, label-free detection of the activities of cells in real-time can be achieved. To address the movement toward spatially resolving cells in cell culture, we developed a sensory system capable of electro-addressing cell location within a microfluidic channel. This simple system allows for real-time cell location, integrity monitoring (of barrier producing cells), and confluency sensing without the need for frequent optical evaluation—saving time. EIS results demonstrate that cells within microfluidic channels can be located between various pairs of electrodes at different positions along the length of the device. Impedance spectra clearly differentiates between empty, sparse, and confluent microfluidic channels. The system also senses the level of cell confluence between electrode pairs—allowing for the relative quantification of cells in different areas of the microfluidic channel. The system’s electrode layout can easily be incorporated into other devices. Namely, organ-on-a-chip devices, that require the monitoring of precise cell location and confluency levels for understanding tissue function, modeling diseases, and for testing therapeutics.

## Introduction

Microfluidics is a powerful platform for cell biological research offering control over cellular microenvironments that effectively simulate dynamic in vivo conditions. When this platform is integrated with electronics, it can extend device functionality and provide precise details about the cellular environment, responses, and movements. For example, electrochemical impedance spectroscopy (EIS) can be coupled with microfluidics. Under the right frequencies, EIS can be used as a non-invasive, label-free method for detecting the activities of cells in real-time and used for extended periods of time to monitor the impedance characteristics of cell cultures within incubators^1^. EIS effectively works by measuring the impedance of electric current by the cell-electrode interface. As cell bodies attach, spread, and cover a sensing electrode, they hinder or impede the exchange of current between the electrode–electrolyte interface. While cells divide, grow, and spread over sensing electrodes, the impedance effectively increases over time. EIS can be used to monitor micromotion^2–4^, cell barrier function^5–9^, cytotoxicity^10,11^, facilitate allergen and compound screening^12,13^, determine cell morphology^14^, and monitor wound-healing^15^.

There is a movement toward spatially resolving cells in cell culture with minimal optical techniques^16–18^. The currently available microfluidic channel impedance-based position sensors only allow for the position of DEP focused or freely flowing single cells and particles to be detected^19,20^. This does not include the sensing of a population of cells grown on and within microfluidic channel surfaces. Other position sensing techniques only examine single cells^21,22^, are limited by small culture chamber volumes (non-microfluidic channels)^16^, require complicated tomography analysis and tools which can suffer from poor spatial resolution^23^ or fail to scan areas on the order of centimeters. Current mapping of the location and confluency of living cells within a microfluidic channel must be done manually by removing cells from the incubator and viewing them under a microscope. Again, impedance can be used to “view” cellular growth and confluency, and, as a bonus, it can also sense local cellular events that cannot be seen through optical observation alone such as ligand binding^6^. An impedance-based position sensor can be valuable for any system involving cellular attachment to a substrate—such as electric cell-substrate impedance sensing (ECIS). ECIS is used for monitoring the attachment, spreading, and integrity of cell monolayers attached to a substrate. Many ECIS studies probe the integrity of barrier producing cells which require confluent cultures, or decent-sized localized cell islands. Conventional ECIS electrode designs use unipolar impedance measurements in which the working electrode is much smaller than the counter electrode. The properties of the small electrode will govern the impedance results due to a higher potential drop occurring near it. One limitation of a typical ECIS system is that only a limited number (between 1 and 1000) of cells can be measured on the working electrode at one time^24^. For a general review of EIS principals and ECIS studies see review articles by Randviir et al. and Hassan et al.^25,26^.

Here, we report on a microfluidic system with an integrated electrode sensor design that allows for an entire cross-section of cells to be electro-addressed and analyzed along the length of a 2 cm microfluidic channel. Impedance-based cell analysis measurements can be done over a narrow or wide range of area along the length of the channel using various electrode combinations. EIS allows for greater characterization of the cells grown within microfluidic channels. Our electrode design allows for a uniform current density, and consistent, sensitive, and repeatable experimental results. The electrodes are intended to accommodate small and large microfluidic channels up to 0.8 cm in width that can be bonded to the chip. Our simple EIS based system saves time by allowing for real-time cell position, integrity, and confluency sensing without the frequent need of a microscope. Optical evaluation of cellular culture is still necessary; however, the reported sensor can be used as a tool to allow continuous analysis and device monitoring without taking cells in and out of the incubator for optical evaluation. In addition, our whole-channel impedance-based position sensor layout can easily be incorporated into other device designs, such as organs-on-chips, that require the precise, continual, and noninvasive cell monitoring benefits that impedance technology has to offer. This is particularly useful for vessel- or blood–brain-barrier-on-a-chip devices that require the examination and testing of confluent layers of barrier producing cells. For that reason, we cultured and tested our microfluidic position sensing system with induced pluripotent stem cell (iPSC)-derived brain microvascular endothelial-like cells (iBMECs) as a proof of concept.

## Results and discussion

### Cellular reprogramming and differentiation

**Figure 1:**
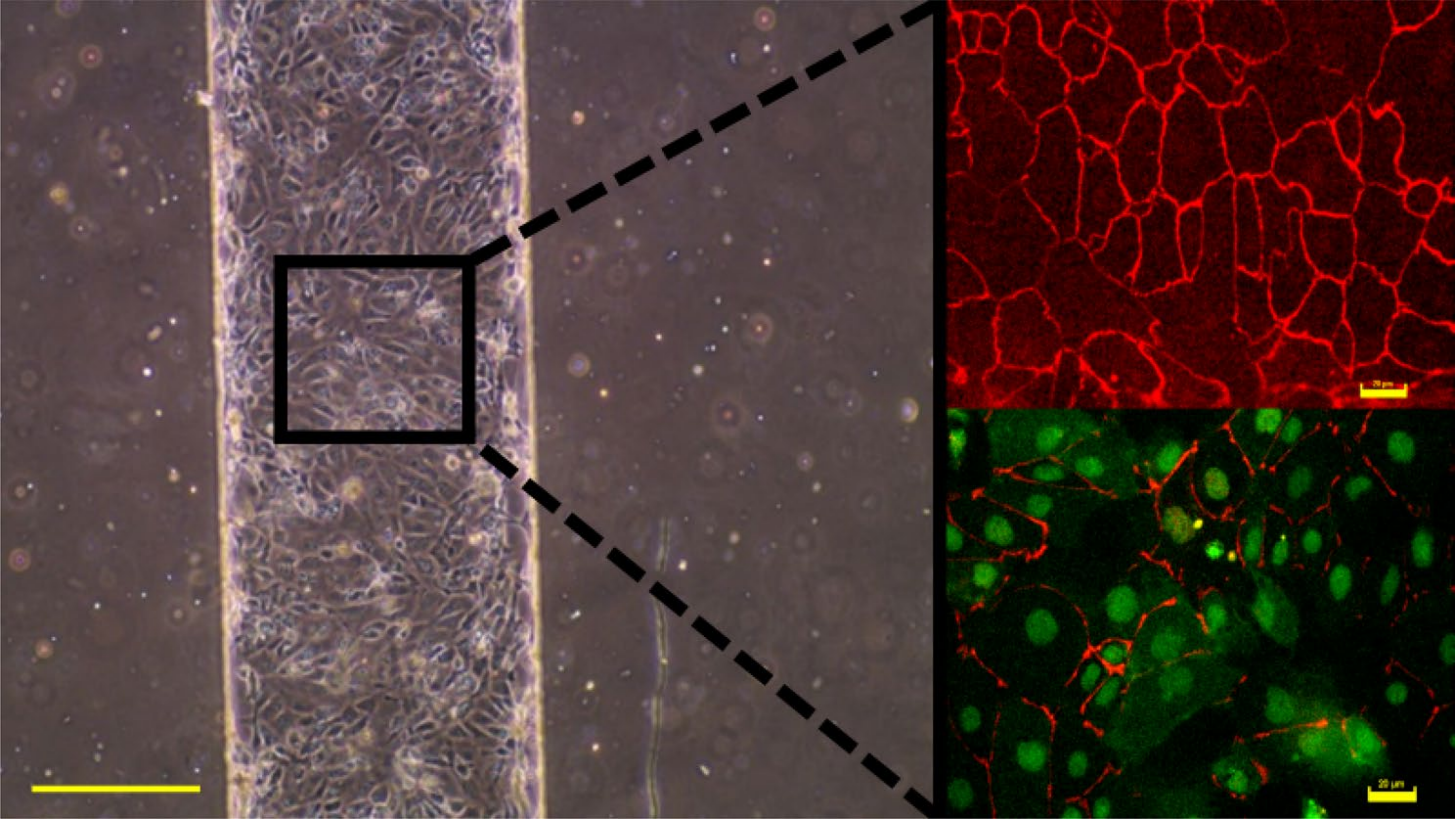
Image of a microfluidic channel containing iBMECs (scale bar 250 µm). The inset contains fluorescent images of iBMEC Zo-1 tight junction proteins (red), nuclei, and GLUT1 glucose transporter proteins covering the cell membrane (green). Inset scale bars are 20 µm.

iPSC-derived brain microvascular endothelial cells were successfully reprogrammed from human omental stromal cells. Figure 1 shows a 500 µm wide channel containing iBMECs after 2 days of growth. The inset containing fluorescent images shows the successful development of tight junctions and blood brain barrier specific transporter proteins present on the surface of iBMEC membranes. Glucose transporter protein 1 (GLUT1) is a highly expressed transporter on the surface of endothelial cells comprising capillaries in the brain, i.e., the blood brain barrier. Immunostaining of the tight junction protein Zonula Occludens 1 (ZO-1) and GLUT1 are important for the qualitative validation of blood brain barrier formation and integrity. Impedance spectroscopy and equivalent circuit modeling can be used for the quantitative analysis of barrier integrity.

### Device design

A cell position impedance-based sensor was successfully integrated into microfluidic channels for the analysis and location of induced brain microvascular endothelial cells. Microfluidic position-sensing devices were made from a 500 µm wide microfluidic channels with four integrated gold electrodes spanning the width of the channels. Gold was chosen for the electrode material due to its inert properties with respect to biological cells and tissue^27–29^. Integrated electrodes were successfully coated with extracellular matrix (ECM), which did not inhibit the overall electrochemical properties of the electrodes, their conductivity, or performance of impedance spectroscopy. Impedance spectroscopy of ECM coated electrodes produces a semi-circle which is indicative of charge transfer reactions occurring between the electrode and the solution proving there is a working electrochemical interface. ECM coating of electrodes does affect the impedance, but not the electrochemical activity of the electrode. ECM is required for iBMEC attachment to substates. We used a commercial reagent from Emulate® to encourage ECM and subsequent cell binding to the device’s glass substrate and planar gold electrodes. A baseline control was created by measuring the impedance of an ECM coated device without cells. The ECM baseline was measured with warm (37 °C) endothelial cell media in order to be compared to experimental measurements with cells present. The EIS profile of empty devices (no cells) containing ECM coated electrodes are presented as solid lines throughout the included figures.

**Figure 2:**
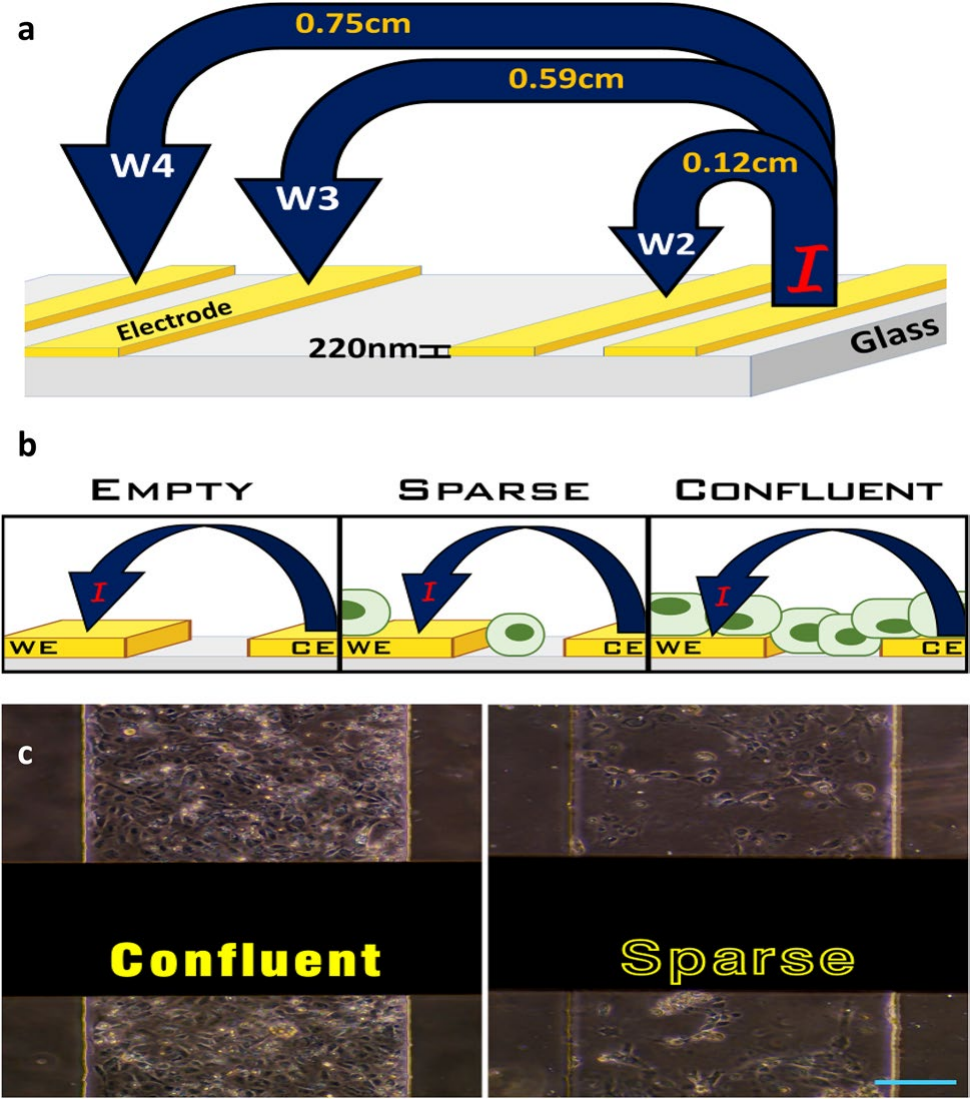
Microfluidic channel images with a schematic of electrodes and impedance workflow. (**a**) Schematic drawing of electrode layout, spacing, height, and electrode functionality with selected electrode probe combinations (W2, W3, & W4). “I” represents the current flowing from the counter (CE) to the working electrode (WE). (**b**) The schematic represents how cellular position and channel confluency are tested. (**c**) Image of confluent and sparse cell coverage of microfluidic channels with integrated gold electrodes (black horizontal bars). Image Scale bar 125 µm.

To detect the presence of adherent cells, various pairs of electrode probes were activated throughout the length of the device’s microfluidic channel. Electrode pair 1&2 correspond to W2; pair 1&3 is W3; 1& 4 is W4; pair 3&4 is W43; and pair 2&3 is labeled as C23 within the reported graph legends. Electrode pairs labeled W2 and W43 have the same distance between them and sometimes served as on chip repeats. Electrode pair W4 represents the furthest distance measured within the system (0.8 cm). The electrode layout, height, interelectrode spacing, and impedance workflow with selected electrode pair combinations (W2, W3, & W4) are represented by the schematic drawing in Fig. 2a. Figure 2b demonstrates the system’s electrode functionality with current (I) flowing from the counter (CE) to the working electrode (WE) for the detection of position and confluency conditions within the microfluidic channel. Images comparing confluent and sparse iBMEC coverage of electrodes and microfluidic channels is presented in Fig. 2c. The black horizontal rectangles in the image are the ECM coated gold electrodes.

### Impedance spectroscopy and interpretation of Nyquist plots

Impedance (Z) is essentially complex resistance as it contains an imaginary component. Unlike resistance, impedance is based on alternating current and its value depends on frequency. Individual elements of a system under investigation can be revealed and characterized by performing a frequency sweep to extract information. Impedance has real (Re(Z)) and imaginary (− Im(Z)) components which are reflected in Nyquist plots as the X and Y axes respectively. The real and imaginary components are plotted against each other at different excitation frequencies. The excitation frequency is not given explicitly but decreases as values move toward the right of the Nyquist plot. Depending on the system being studied, Nyquist plot traces can take on the shape of a semi-circle, a semi-circle with a tail, a straight line, etc. The trace profiles can provide information on the frequency-dependent impedance characteristics of an investigated system such as reaction kinetics, charge/electron transfer, diffusion, capacitance, solution resistance, etc.

**Figure 3:**
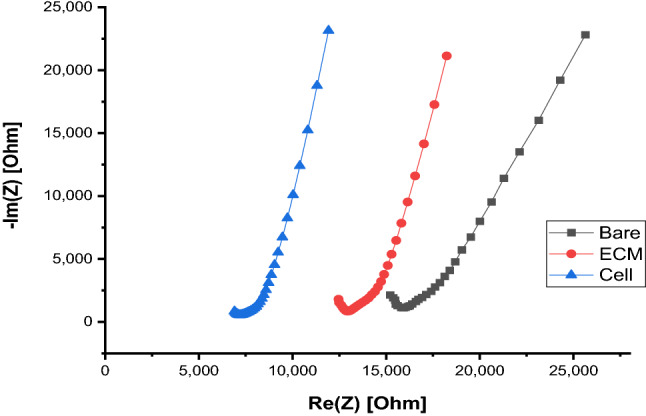
A comparison between bare, ECM coated, and cell covered ECM-gold electrodes. As electrode coverage increases, there is a decrease in impedance for the reported electrode design.

**Figure 4:**
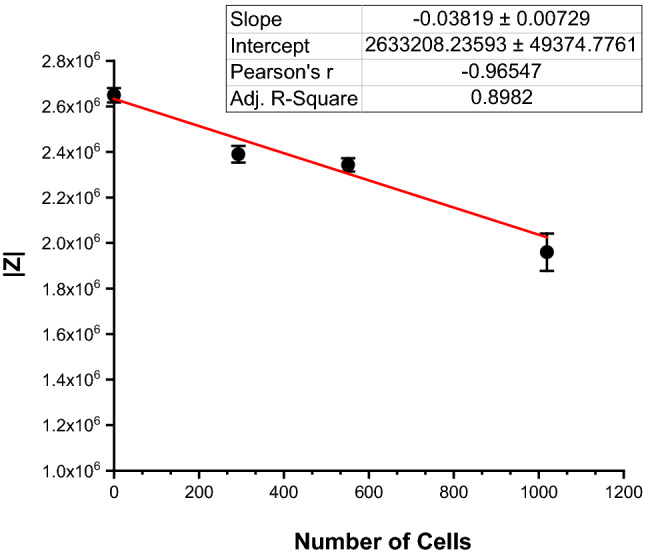
Correlation of absolute impedance to the number of cells.

To obtain more detailed information about the cellular presence and dispersal conditions within the channel, various electrode probe pairs were examined to detect the location, presence or absence, and relative amounts of cells attached to the substrate. Repeatable patterns have been found for the presence of cells at different locations along a microfluidic channel. The Nyquist impedance plot traces in Fig. 3 compare bare gold, ECM coated (no cells), and cell covered electrodes (ECM coated gold electrodes with cells). For our microfluidic parallel coplanar electrode layout, Nyquist plot traces shift to the left (having a lower absolute impedance) as electrode coverage increases. This is seen by tracking the checkmark-like traces (knee position) along the Re(Z) x-axis in Fig. 3. A linear correlation was found between the number of cells and absolute impedance with a regression slope of − 0.03819 ± 0.00729, an R-squared value of -0.96547, and a *p*-value of ≤ 0.05 (Fig. 4). Impedance decreases with an increase in cell number for the parallel coplanar electrode design. Impedance spectra was able to discriminate between the range of fully confluent microfluidic channels or sections, sparse channels or sections, and channels where cells have detached from the substrate.

**Figure 5:**
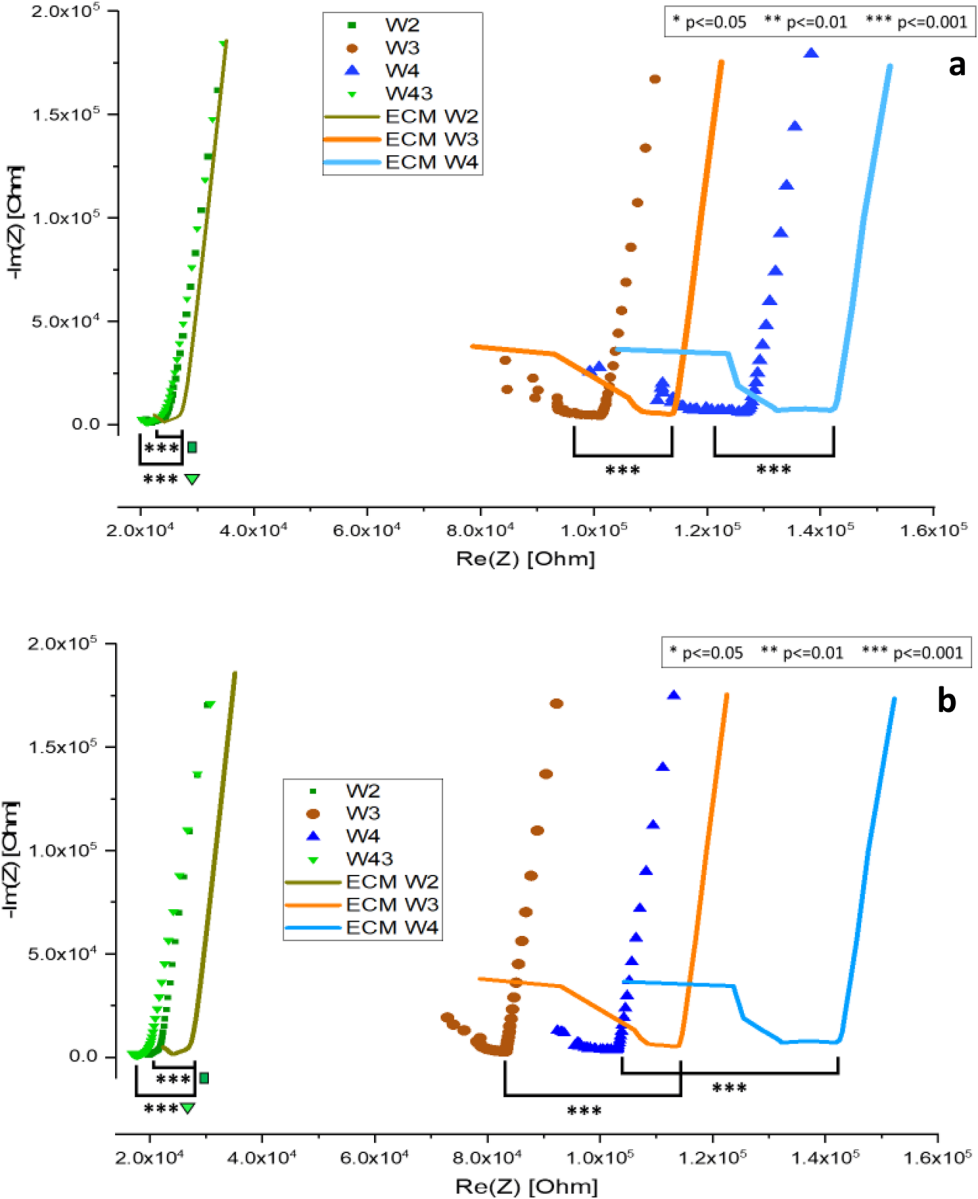
Nyquist plots of two position-sensing microfluidic devices. Legend W2, W3, W4, and W43 are the labels for the cell containing device electrode probe combinations, 1 & 2, 1 & 3, 1 & 4, and 3 & 4 respectively. ECM labels correspond to ECM coated electrode probe combinations in devices without cells (controls).

**Figure 6:**
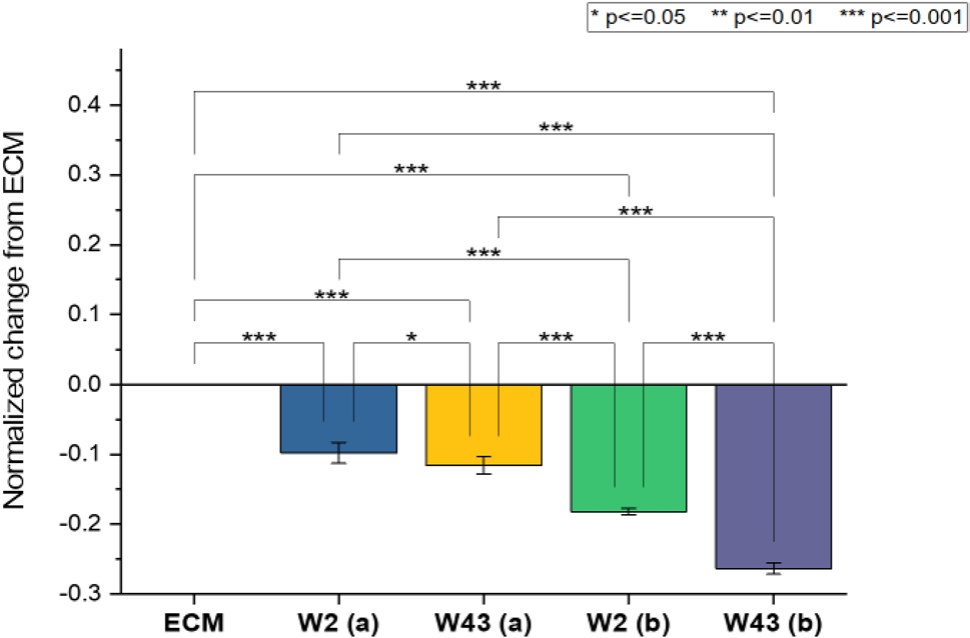
Graph of W2 and W43 electrode pairs from devices “a” and “b” in Fig. 5a, b respectively, normalized to ECM.

Figure 5 is a Nyquist impedance plot of iBMECs grown in two separate 500 µm wide microfluidic position-sensing devices. The EIS profile of the ECM control (ECM coated electrodes without cells present) is presented as solid lines. Following the same trend as Fig. 3, the presence of more cells will shift the knee position of the Nyquist plot traces to the left toward higher frequencies (M-kHz) indicating a decrease in charge transfer resistance. The relative confluency of cells between each pair of electrodes can be determined by the left-shift of cellular plot traces compared to ECM control traces within the Nyquist plot. As expected, the impedance trace profiles of the cells do not align with the ECM controls indicating the presence and dispersal of attached cells throughout the microfluidic channel (Fig. 5a, b). Electrode pairs 1&2 (W2) and 3&4 (W43) have the same distance between electrode probes and are positioned at opposite ends of the device. Figure 6 is a comparison of normalized absolute impedance for electrode pairs W2 and W43 for devices “(a)” and “(b)” depicted in Fig. 5a, b respectively. Negative values reflect the left-shift of cell covered electrodes versus ECM controls on the Nyquist plot Re(Z) axis. For the first device (Fig. 5a), the EIS traces for electrode pairs W2 (green square) and W43 (green triangle) almost align, reflecting a similar amount of confluent cells present between each electrode probe pair. The normalized data in Fig. 6 reveals both W2 and W43 for device (a) have respective averages of -0.09789 ± 0.01513 and -0.11558 ± 0.01207 with significance of *p* ≤ 0.05 between them.

For the second device represented in Fig. 5b, a comparison of cell covered electrode pairs W2 and W43 versus their ECM control shows that they are shifted to the left more than the W2 and W43 traces in Fig. 5a. The Nyquist plots along with normalized change comparisons in Fig. 6 reflect that there are more cells between the W2 and W43 electrode pairs in the device (b) than device (a). Another example of the relative quantification of cells occurs when the distance between W3 (dark orange circles) and ECM W3 (orange solid line) are examined for both devices. There are more cells detected in Fig. 5b W3 probe pair versus Fig. 5a W3 probe pair as indicated by the distance between traces on the Real (Z) x-axis**.** This is also validated by comparing the normalized changes in impedance of W3 for both devices where a *p* value of ≤ 0.001 was found**.**

**Figure 7:**
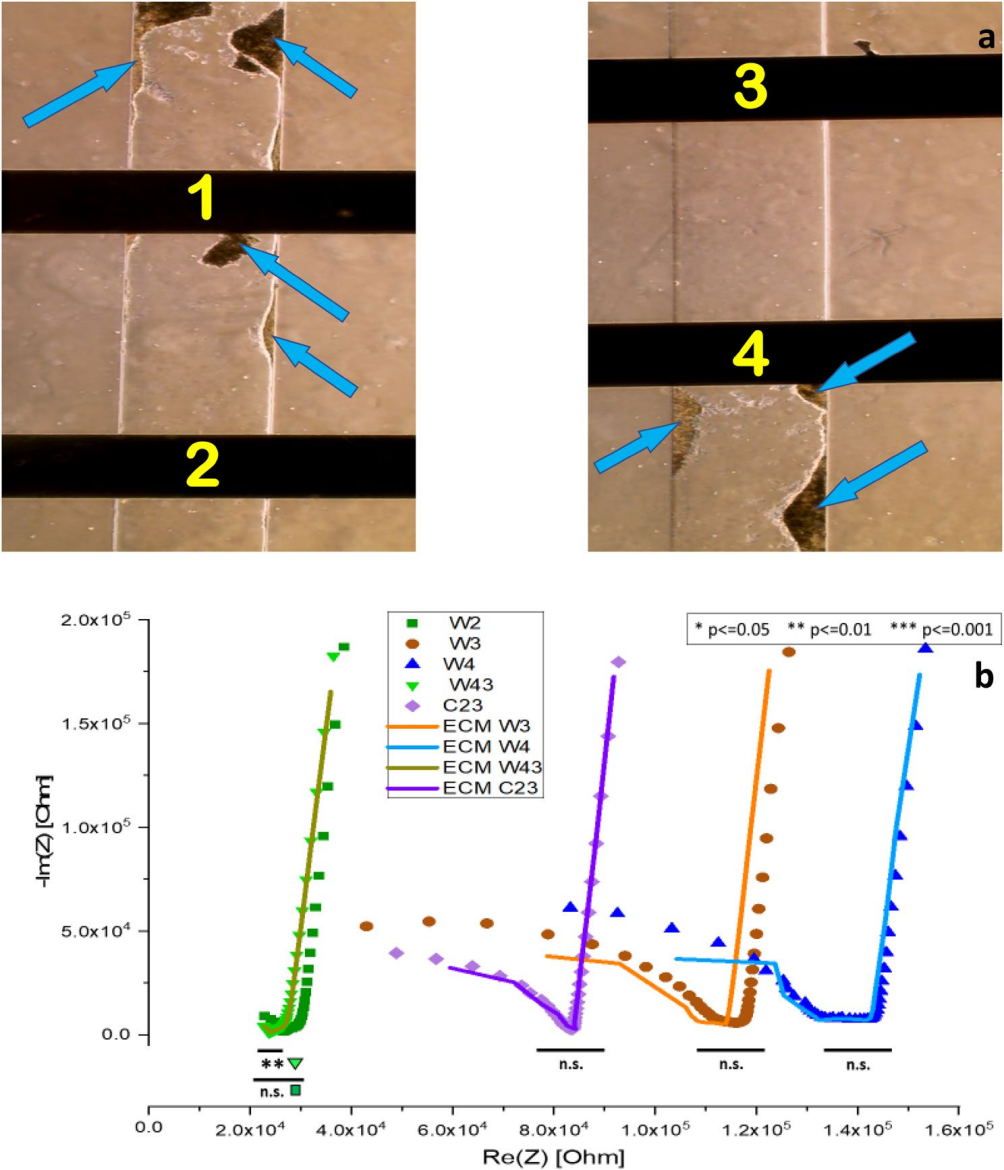
Image of device with unattached cells and its corresponding Nyquist plot. (**a**) Image showing four electrodes along the channel of one microfluidic device. Arrows point to areas where cells have detached from the substrate and aggregated together. The legend label C23 corresponds to electrode pair 2 & 3.

Detached and sparse cell culture within microfluidic devices can be detected with the presented position sensing system. Figure 7 provides an example of EIS results when cells detach from the ECM coated channel substrate, leaving cell aggregates along the channel edges. The images and the corresponding Nyquist plot in Fig. 7 demonstrate the condition and trace profiles of the microfluidic channel when this situation occurs. The cell trace profiles align with the ECM control trace profiles—revealing little to no cell attachment (Fig. 7b). This is indicated by *p* values for the various electrode combinations along the length of the channel.

**Figure 8:**
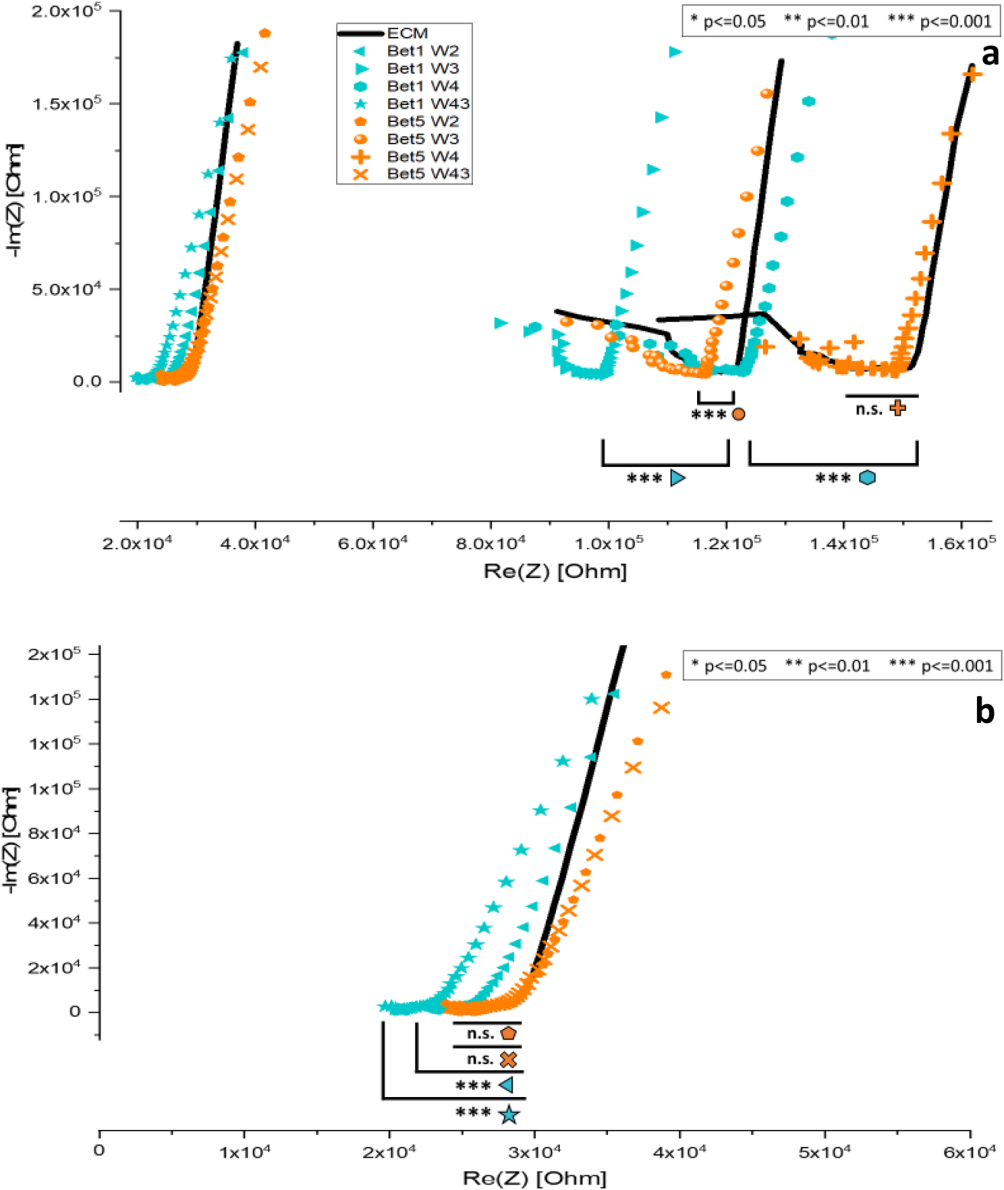
(**a**) Nyquist plot comparing two devices (Bet1 and Bet5) on day 1 of incubation. (**b**) A closeup of the lower Real Impedance (x-axis) region showing electrode probe pairs W2 and W43 for both devices compared to the ECM control (black). ECM traces are black in this figure to help with the comparison of Bet1 and Bet5. The order of the ECM controls is the same as in previous figures.

Multiple devices can be compared at the same time to assess their level of confluency and the location of cell islands. Figure 8 is a Nyquist plot comparing two devices (Bet1 and Bet5). ECM traces are colored black in this figure to make the comparison between Bet1 and Bet5 clearer. The order and the position of the ECM controls on the Re(Z) x-axis are the same as in other figures. Both Bet1 and Bet5 devices were seeded on the same day (day 0) with the same batch and concentration of cells. Upon observation the next day (day 1), the Nyquist plot shows device Bet1 (turquoise marker) is populated with more adherent cells than device Bet5 (orange marker) based on the statistically significant shifts from ECM. Bet5 mostly aligns and matches the ECM control profiles for electrode pairs, W2, W43, and W4; however, there is an indication of cells dispersed between electrodes 1&3 (W3). Deducting the information from electrode pairs W2 (1&2) and W3 (1&3), one can determine that cells are dispersed between electrodes 2&3 using the Nyquist plot results. P-values reveal device Bet5 is generally not significantly different from ECM baseline control overall. However, Bet1 and Bet5 are significantly different from each other on day 1 of incubation with *p* ≤ 0.001 for all electrode pairs compared between the two devices.

## Conclusions

To address the movement toward spatially resolving cells in cell culture, we successfully developed and demonstrated an impedance-based position sensor system capable of electro-addressing cell location, assessing confluency, and quantifying the relative amount of cells present within a microfluidic channel. This system was integrated with four bands of gold electrodes spanning the width of 500 µm wide microfluidic channels. These electrode probes were placed at different distances along the length of the channel to probe for cells. We utilized iBMECs as proof of concept for assessing the position and confluency of cells that, in nature, are required to produce a continuous barrier.

EIS results demonstrate that the locations of cells can be addressed using various pairs of electrodes at different positions along the length of the device. Impedance spectra distinguishes between empty, sparse, and confluent microfluidic channels. The electrodes can sense the confluency of cells located at different positions allowing for the relative quantification of cells between electrode pairs, especially when comparing multiple devices. Moreover, the electrode design can be easily integrated into other microfluidic engineered constructs such as organ-on-a-chip devices. A modified version of the device design herein can be used within blood vessel- or blood–brain–barrier-on-a-chip devices to probe the confluency, barrier integrity, and the best area for transendothelial or ECIS studies. To use our particular electrode layout, other groups would need to establish a baseline with the specific ECM coatings required for their specific cell experiments. This can be done on a non-cell containing uncoated device and ECM coated device. The devices must be treated as if cells were present, meaning they must be kept in an incubator and tested with the specific cell media used for cellular experiments. The baseline devices must also be tested for the same amount of days as the cell experiments. For example, if cells are grown within a device for three days and tested on day three, baseline ECM profiles should also be tested after 3 days of incubation. Theoretically, multiple cellular cultures can remain inside an incubator and be tested on demand with a portable multichannel potentiostat.

Overall, this simple microfluidic channel system allows for real-time electro-addressing of cell position, the examination of cellular attachment, confluence, and barrier integrity testing (for barrier producing cells) without the use of initial or frequent microscopic evaluation—saving time. Modified versions of this system will prove to be valuable in microfluidic cell culture and organ-on-a-chip constructs that necessitates precise cell location and cellular confluency levels for the modeling of diseases, testing of therapeutics, and for investigating basic research questions such as cell/tissue function and communication.

## Materials and methods

### Fabrication of microfluidic chips

#### Channel mold fabrication

Microfluidic channels were designed using CleWin4 CAD software and printed as a photomask through CAD/Art Services, Inc. Channels were fabricated using standard photolithography techniques. Briefly, a four-inch silicon wafer was spin coated with SU-8 3050 photoresist and soft-baked at 95 °C for 15 min. The spin coated wafer was then covered with a photomask containing the microfluidic channel designs. The wafer and photomask were exposed to UV light for patterning. The silicon wafer was subsequently baked at 65 °C for one minute and 95 °C for 5 min before development with AZEBR developer, according to the MicroChemicals processing guidelines, to achieve channel feature heights of 50 µm.

#### Electrode fabrication

Electrodes were designed on CleWin4 software and the mask was printed through CAD/Art Services, Inc. Electrodes were fabricated using a photolithography lift-off technique and E-beam evaporation. Briefly, a four-inch borosilicate glass wafer is spin-coated with image reversal photoresist AZ-5214E to achieve a thickness of 1.6um. The borosilicate glass wafer is then soft baked and exposed to UV light for 10 s, followed by a postexposure bake and UV flood exposure for 150 s according to Merck technical guidelines through MicroChemicals. The borosilicate wafer was immersed in MIF726 developer to bring out the electrode design features. The wafer was then deposited with 20 nm of titanium followed by 200 nm of gold. After metal deposition, the wafers were subsequently soaked in acetone for an hour to complete the lift-off process exposing individual electrodes. The glass wafers were diced into individual pieces (electrode chips).

#### Device assembly

Microfluidic channels were made using the silicon molds (described in the channel mold fabrication section) and a polydimethylsiloxane (PDMS) replica molding process. The process requires 10:1 pdms base to curing agent ratio to be mixed thoroughly, poured onto silicon molds and degassed in a vacuum chamber. The silicon molds were then placed into an 80˚C oven for a minimum of 2 h. After the pdms solidified and cooled, microfluidic channels were cut and removed from the mold. For device assembly, PDMS channels and electrode chips were plasma treated, aligned, and placed into contact with each other for permanent bonding. To prepare for cell seeding, device channels were sterilized and coated with Emulate® reagent. The Emulate® coated device channels were exposed to UV light for 30 min and then rinsed with PBS. The device channels were subsequently coated with extracellular matrix (ECM) containing a 4:1 mixture of 1 mg/mL of human placenta type IV collagen and 1 mg/mL fibronectin (Sigma-Aldrich) and placed into a 37 °C incubator overnight.

### iPSC-derived iBMEC cellular reprogramming, differentiation, and culture

Induced pluripotent stem cell (iPSC)-derived brain microvascular endothelial-like cells (iBMECs) were reprogrammed from human omental stromal cells gifted by Dr. Rivka Ofir of Ben Gurion University of the Negev. To differentiate iPSCs into iBMECs, iPSCs were passaged over Matrigel in NutriStem™ medium for two to three days for cell culture expansion up to 25–30% confluency (a density of 2–3 × 10^5^ cells/well). For the following six days, cells were changed to an unconditioned medium lacking basic fibroblast growth factor (bFGF). Two days following this, human endothelial serum-free medium (hESFM; Life Technologies) supplemented with 20 ng/ml (bFGF, Peprotech) and all-trans retinoic acid (RA, 10 mM; Sigma), was added. Cells were then gently dissociated and seeded into PDMS channels after being incubated with Accutase for 30 to 35 min. iBMECs were then grown without bFGF and RA in endothelial cell medium.

### Device seeding and electrochemical impedance spectroscopy (EIS)

Microfluidic position sensing devices were seeded with induced pluripotent stem cell (iPSC)-derived brain microvascular endothelial-like cells (iBMECs) at 14–20 × 10^6^ cells/mL. The high density is necessary for the size of the channel and to ensure a complete monolayer of cell coverage for confluency testing. Cells were grown and monitored until they reached desired confluency. Following seeding, the establishment of a monolayer was visually monitored. Lower densities were previously shown to result in suboptimal cell coverage^30,31^. Unattached cells were washed away. Experiments were performed on six independent devices. Presented Nyquist plots are representative of these repeats. Devices termed “ECM control” are empty devices (devoid of cells) with ECM coated gold electrodes. The empty device serves as a baseline and was measured up to four times (with the shortest electrode distances) and measured on different incubation days. The baseline devices were treated as if cells were present. They were kept in an incubator for the same amount of time as the devices containing cells and tested with the same cell media. Impedance measurements were taken 1–3 days after seeding. Potentio Electrochemical Impedance Spectroscopy was performed with a VSP-300 potentiostat from Biologic Scientific instruments (Cliax, France). Impedance spectra was analyzed with the provided EC-Lab software v11.32. Impedance measurements were taken between different pairs of gold electrodes along the length of the microfluidic channel with frequencies between 0.5 HZ and 1 MHz and a modulation (AC) voltage of 50 mV. Impedance measurements were taken in an incubator within a faraday cage at 37 °C for 1.5 min per electrode combination.

Impedance data was normalized to corresponding electrodes coated with ECM but not seeded with cells for each electrode pair by dividing cell absolute impedance values by ECM absolute impedance values and subtracting one from the quotient. Negative values (a left shift from ECM controls in Nyquist plots) indicate the presence of cells. Any positive values, corresponding to traces shifted to the right of ECM controls, were set to zero because traces moved toward bare gold electrode trace profiles. We suspect that the system is detecting areas where some cells have unattached AND removed ECM in the process. All data sets were tested using one-way ANOVA with Tukey's post-hoc test for multiple comparisons.

### Immunocytochemistry and imaging

After two days of growth and EIS measurements, cells were fixed for staining. They were washed twice with Dulbecco's Phosphate-Buffered Saline (DPBS), then fixed in 4% paraformaldehyde for 20 min at room temperature. 1% bovine serum albumin combined with 0.1% triton x-100, was used as a blocking solution for membrane permeabilization. Cells were then incubated in a blocking solution with primary antibody Glucose transporter 1 (GLUT-1) (1:300, Thermo Fisher, MA5-11315); Zonula Occludens 1 (ZO-1) (1:100, Thermo Fisher, Zy-617300), overnight at 4 °C. After washing with DPBS, the cells were incubated for two hours at room temperature with fluorescently labeled secondary antibodies (1:500; Invitrogen). Samples were imaged using a ZEISS LSM 900 microscope.
